# Tuberculose péritonéale pseudo tumorale: à propos d’une série de 14 cas

**DOI:** 10.11604/pamj.2022.43.130.35899

**Published:** 2022-11-09

**Authors:** Allae Eddine Bouchaib, Jihad Drissi, Abdellah Babahabib, Moulay El Mehdi Elhassani, Jaouad Kouach

**Affiliations:** 1 Service de Gynécologie-Obstétrique, Hôpital Militaire d’Instruction Mohamed V, Rabat, Maroc

**Keywords:** Tuberculose péritonéale, cancer de l’ovaire, carcinose péritonéale, cœlioscopie, Peritoneal tuberculosis, ovarian cancer, peritoneal carcinosis, laparoscopy

## Abstract

La forme pseudo-tumorale de la tuberculose péritonéale est peu fréquente, mais son incidence reste considérable en zone d´endémie. Dans le but d´illustrer le problème de diagnostic différentiel avec le cancer de l´ovaire vu la grande similitude radio-clinique entre ces deux entités pathologiques, nous avons réalisé une étude rétrospective au service de gynécologie obstétrique de l´Hôpital Militaire d´Instruction Mohammed V de Rabat, portant sur 14 cas (n= 14) de tuberculose péritonéale pseudo tumorale. Toutes les autres localisations extra pelviennes ont été exclues, L´âge moyen de nos patientes est de 33,4 ans avec un maximum de cas dans la tranche 16-40 ans: 71% (n=10/14). Le tableau clinique habituel de cette forme particulière de tuberculose péritonéale est celui d´une douleur abdominale: 100% (n=14/14) associée à une masse abdominopelvienne: 71% (n=10/14) et ascite: 64% (n=09/14) mimant en tout point une carcinose péritonéale notamment d´origine ovarienne, d´autant plus que les deux pathologies évoluent dans un contexte d´altération de l´état général. Le diagnostic fait appel aux explorations invasives par cœlioscopie: 35% (n=05/14) ou laparotomie: 57% (n=08/14) avec biopsies. En effet seule la preuve histologique permet de réconforter le diagnostic dans la majorité des cas. La prise en charge thérapeutiques de nos patientes a été constituée de deux volets: un traitement médical selon le programme national de lutte anti tuberculeuse et le traitement chirurgical. Le recours aux explorations invasives est souvent inéluctable avant d´instaurer tout traitement anti bacillaire. L´évolution sous traitement spécifique est favorable, le pronostic de fertilité reste engagé chez la femme jeune.

## Abstract (en)

Pseudotumoral peritoneal tuberculosis is uncommon, but its incidence is high in endemic areas. Given the great radioclinical similarity between pseudotumoral peritoneal tuberculosis and ovarian cancer, we conducted a retrospective study in the Department of Obstetrics and Gynaecology of the Military Hospital of Instruction Mohammed V in Rabat, involving 14 cases (n= 14) of pseudotumoral peritoneal tuberculosis in order to illustrate the problem of differential diagnosis. All other extra-pelvic locations were excluded, the average age of our patients was 33.4 years with a maximum of cases in the 16-40 years group: 71% (n=10/14). Common clinical symptoms of this particular form of peritoneal tuberculosis were abdominal pain: 100% (n=14/14) associated with abdominopelvic mass: 71% (n=10/14) and ascites: 64% (n=09/14) mimicking peritoneal carcinosis of ovarian origin, especially since both pathologies progressed in a context of impaired general condition. Diagnosis was based on invasive laparoscopic examinations: 35% (n=05/14) or laparotomy: 57% (n=08/14) with biopsies. Indeed, only histological examination can help to establish definitive Corriger diagnosis, in the majority of cases. Therapeutic management of our patients was based on medical treatment, according to the national tuberculosis control program, and surgical treatment. The use of invasive explorations is often unavoidable before initiating any anti-bacillary treatment. Patients´ outcome under specific treatment is favorable, the prognosis of fertility is engaged in young women.

## Introduction

La tuberculose est une maladie infectieuse curable. Sa prévalence a connu une recrudescence dans le monde entier ceci s'explique par l'immunodépression liée à l'infection par le VIH [1]. La tuberculose (TB) péritonéale est rare; elle occupe le quatrième rang des localisations extra-pulmonaires, elle est de diagnostic souvent difficile car des caractéristiques cliniques et radiologiques qui manquent de spécificité [2]. Dans sa forme pelvienne pseudo tumorale elle présente de nombreuses similitudes cliniques, biologiques et radiologiques avec le cancer de l´ovaire qui constitue le principal diagnostic différentiel [3,4]. Le diagnostic de certitude fait appel aux explorations invasives à savoir la cœlioscopie voire la laparotomie avec biopsies. En effet, seule la confirmation histologique justifie l´instauration du traitement anti bacillaire. L´objectif de ce travail est de contribuer à l´étude des différents aspects clinico-biologiques, radiologiques, endoscopiques et thérapeutiques des formes pseudo tumorales des tuberculoses péritonéales en rapportant l´expérience du service de gynécologie-obstétrique de l´Hôpital Militaire d´ Instruction Mohamed V de Rabat en vue Corriger d´illustrer le problème de diagnostic différentiel avec le cancer de l´ovaire.

## Méthodes

**Conception et mise en place de l'étude**: il s´agit d´une étude rétrospective étalée sur une période de dix ans de Mars 2010 au mois de Mai 2020 menée au service de Gynécologie-Obstétrique de l´Hôpital Militaire d´Instruction Mohamed V de Rabat ayant inclus toutes les formes pseudo tumorales de tuberculose péritonéale et qui simulent un cancer ovarien vu que le Maroc est un pays endémique pour cette maladie.

**Population étudiée**: un total de 14 cas de tuberculose péritonéale pseudo-tumorale a été colligé. Pour toutes nos patientes, le diagnostic de tuberculose a été confirmé par la présence de granulomes épithélio-giganto-cellulaire avec ou sans nécrose caséeuse à l´examen anatomopathologique des biopsies péritonéales , ont été exclues toutes les autres localisations extra pelviennes notamment les tuberculoses mammaires, vaginales et vulvaires, ainsi que les autre localisations pelviennes non pseudo tumorales notamment les hydrosalpinx isolés, les synéchies tuberculeuses et les infertilités d´origine tuberculeuse.

**Collecte de données**: une fiche de référence a été utilisée pour collecter les données au près des dossiers archivés pendant une période de 10 ans au sein du service de gynécologie obstétrique à l´hôpital militaire Mohammed 5. Nous avons précisé pour chaque cas les caractéristiques épidémiologiques (âge, antécédents personnels et familiaux de TB), cliniques (existence d´une TB évolutive, délai de consultation par rapport au premier symptôme, plaintes fonctionnelles, données de l´examen physique), biologiques (données cytochimiques et bactériologiques du liquide d´ascite, vitesse de sédimentation (VS)) et radiologiques (anomalies de la radiographie thoracique, les résultats de la tomodensimérie (TDM) abdomino-pelvienne). Nous avons également colligé le résultat et la méthode d´exploration de la cavité péritonéale (laparotomie ou cœlioscopie) ainsi que les aspects évolutifs après traitement.

## Résultats

**Caractéristiques générales**: l´âge moyen de nos patientes est de 33.4 ans avec un maximum de cas dans la tranche 16-40 ans: soixante-onze pour cent (71% (n=10/14)). Toutes étaient issues d´un bas niveau socio-économique. Seul 5 patientes avaient des antécédents personnels de tuberculose; une a été opérée pour tuberculome cérébrale et l´autre avait une tuberculose pulmonaire, alors que la notion de contage tuberculeux était présente chez 3 de nos patientes. Un cas de tuberculose pulmonaire évolutive a été mis en évidence. Le délai moyen entre l´apparition des premiers symptômes et la date de la consultation était de 4 mois (extrêmes: 3 à 7 mois Espace).

**Caractéristiques cliniques**: outre la douleur abdomino-pelvienne le tableau clinique était dominé par les signes d´imprégnation tuberculinique notamment l´amaigrissement et par l´installation d´une ascite. Les différentes manifestations cliniques sont détaillées dans le Tableau 1. La mesure de la vitesse de sédimentation a été pratiquée dans 06 cas où elle a été élevée. L´étude du liquide d´ascite chez les 09 patientes qui présentaient une ascite a trouvé dans tous les cas un liquide exsudatif lymphocytaire dépourvu de cellules malignes. Le dosage de la CA125 est revenu élevé dans 13cas/14 avec une valeur moyenne de 352.5UI/ml (extrêmes 112-500). La radiographie thoracique a mis en évidence un nodule pulmonaire dans un cas. La TDM abdomino-pelvienne a été réalisée dans 09 cas et l´IRM chez une seule patiente. Les principaux signes radiologiques retrouvés sont résumés dans le Tableau 2.

**Tableau 1:** manifestations cliniques de la tuberculose péritonéale

Manifestation clinique	Nombre
Fièvre	4
Amaigrissement	11
Douleur abdominale	14
Ascite	9
Masse abdomino-pelvienne	10
Signes de compression digestive	4
Troubles menstruels	2

**Tableau 2:** manifestations radiologiques de la tuberculose péritonéale

Signe radiologique	Nombre
Ascite	9
Formation à composante mixte tissulaire et kystique	8
Formation kystique cloisonnée	8
Epaississement péritonéal	8
Epaississement mésentérique et/ou épiploïque	14

**Gestion et résultats**: l´exploration du péritoine a été faite chez 13 patientes, par cœlioscopie dans 05 cas et par laparotomie dans 08 cas. Une patiente chez qui le diagnostic de tuberculose était retenu par présomption est décédé en préopératoire dans un tableau d´hémorragie digestive par rupture de varices œsophagiennes. Les différents aspects macroscopiques sont décrits dans le Tableau 3. L´étude histologique des prélèvements biopsiques a objectivé des granulomes épithélio-giganto-cellulaires avec nécrose caséeuse dans 10 cas et sans nécrose caséeuse dans 4 cas. La prise en charge thérapeutiques de nos patientes a été constituée de deux volets: un traitement médical selon le programme national de lutte anti tuberculeuse et un traitement chirurgical comportant les gestes suivants: libération des adhérences, drainage chirurgical d´un abcès tubo-ovarien, mise à plat d´un pyosalpinx, annexectomie. Les suites postopératoires étaient simples chez 12 patientes, une patiente a présenté une éventration en postopératoire nécessitant une reprise chirurgicale. Dans les autres cas, sous traitement anti bacillaire bien conduit, l´évolution a été favorable marquée par une amélioration clinique (défervescence thermique, reprise pondérale, disparition de l´ascite et de la douleur abdominale), biologique (normalisation du bilan inflammatoire et négativation du dosage du CA125) et radiologique.

**Tableau 3:** aspect macroscopique du péritoine au cours de la tuberculose péritonéale

Aspect macroscopique	Nombre
Masse pseudo tumorale	13
Granulations blanchâtres	12
Adhérences péritonéales	12
Hyperhémie	14

## Discussion

L´objectif de ce travail est de contribuer à l´étude des différents aspects clinico-biologiques, radiologiques, endoscopiques et thérapeutiques des formes pseudo tumorales des tuberculoses péritonéales en rapportant l´expérience du service de gynécologie-obstétrique de l´Hôpital Militaire de Rabat en vue d´illustrer le problème de diagnostic différentiel avec le cancer de l´ovaire. L´incidence de la tuberculose au Maroc est de 29 000 à 30 000 nouveaux cas par an avec une prévalence chez le sujet jeune de 21-45 ans [3,4]. Dans notre étude l´âge moyen est de 32,4 ans, 3 de nos patientes sont âgées de moins de 21 ans et 3 de plus de 45 ans. La TBP représente 1 à 2% de toutes les localisations et 31 à 58% des localisations abdominales. La forme pseudo tumorale est relativement rare elle représente 15% des localisations pelviennes [4]. Plusieurs facteurs favorisants sont relevés: le VIH, la corticothérapie prolongée, le bas niveau socio-économique, le traitement par immunosuppresseurs, la BCG-thérapie [5,6]. Dans notre série les antécédents de tuberculose ont été notés chez 5 patientes. La tuberculose péritonéale est une maladie d´évolution subaigüe; le délai moyen de consultation est de 2 à 4 mois [7] comme il a été noté dans notre série.

Le tableau clinique habituel est celui d´une ascite avec douleurs abdominales et signes d´imprégnation tuberculiniques. D´autres signes devront être recherchés à type de troubles menstruels, troubles digestifs ou urinaires. Une association à d´autres localisations notamment pulmonaire ou digestif doit être recherchée, cependant absente dans 30 à 50% des cas [8]. Dans notre série outre les algies pelviennes, la distension abdominale, l´ascite et les signes d´imprégnation tuberculiniques présents chez la quasi-totalité de nos patientes, deux ont eu des troubles menstruels et trois des signes de compression urinaire ou digestif. Une masse abdominopelvienne a été retrouvée chez 10 patientes.

Le dosage du CA 125 est peu spécifique ; Sous traitement anti bacillaire son taux chute puis se normalise au bout de 1 à 2 mois [8]. Dans notre série, 13 patientes ont bénéficié du dosage du CA125 avec des taux variant de 112 à 500 UI/L. L´intradermoréaction à la tuberculine peut orienter le diagnostic mais avec de nombreux faux négatifs (15à60%) [8]. Le dosage de l´activité de l´adénosine désaminase (ADA) au seuil de 30UI/L, a une sensibilité de 96%, une spécificité de 98%. Il est peu coûteux, rapide, son dosage est recommandé selon la société française de pneumologie [9]. Le dosage de l´interféron gamma demeure limité en routine [10,11]. Le test de la Polymérase Chain Réaction(PCR) et la réaction d´amplification génique par LCR permettent d´isoler le BK en 24 à 48h mais leur coût est élevé avec une sensibilité réduite [4]. L´échographie abdominale trouve une ascite dans 45 à 100% des cas souvent libre mais parfois cloisonnée. D´autres signes peuvent s´y associer permettront d´évoquer le diagnostic sans pouvoir écarter le diagnostic de carcinose péritonéale [12]. Dans sa forme pseudo tumorale, la tuberculose pelvi péritonéale réalise l´aspect d´image pelvienne solidokystique hétérogène pouvant fistuliser aux organes de voisinage [8]. Dans notre étude l´échographie a été réalisée chez 13 patientes avec des images faisant suspecter un cancer de l´ovaire. La TDM est plus pertinente ; elle évoque l´origine tuberculeuse devant la présence d´adénomégalies hypodenses se rehaussant en périphérie [12]. Devant un tableau radio-clinique évocateur d´autres localisations notamment pulmonaires devront être recherchées. Les moyens d´imagerie permettraient d´orienter une biopsie transvaginale ou percutanée surtout en cas d´épaississement du grand épiploon [9]. Dans notre étude la TDM a été pratiquée chez 11 patientes avec des images radiologiques faisant suspecter un cancer ovarien.

Les explorations invasives à savoir la cœlioscopie ou la laparotomie occupent une place prépondérante dans l´affirmation du diagnostic, elles permettent de réaliser des biopsies [4]. L´exploration de la cavité péritonéale trouve des granulations blanchâtres, des nodules péritonéaux, une hyperhémie du péritoine, des adhérences péritonéales filamenteuses, une agglutination des anses intestinales. A l´opposé les nodules de carcinose péritonéale sont ombiliqués, rétractés, de taille variable, avec un péritoine non inflammatoire. La découverte d´une masse notamment ovarienne conduit dans la majorité des cas, à un traitement radical type annexectomie [2]. L´étude histologique des prélèvements biopsiques objective des granulomes épithéliogigantocellulaires avec habituellement une nécrose caséeuse [7]. Dans notre série, 13 de nos patientes ont bénéficiées d´une exploration invasive avec biopsie et examen extemporané confirmant la présence de granulomes épithélio-giganto-cellulaires avec nécrose caséeuse chez 12 de nos patientes, et sans nécrose caséeuse chez 2 d´entre elles avec absence de cellules tumorales. Le traitement de la tuberculose péritonéale repose sur une quadrithérapie anti bacillaire: rifampicine, isoniazide, pyrazinamide, ethambutol pendant 2 mois puis une bithérapie: rifampicine et isoniazide pendant 4 mois. La chirurgie s´avère nécessaire en cas de signe de compression digestive ou urinaire ou dans les formes fistulisées [1]. Certains auteurs préconisent d´adjoindre la corticothérapie dans le but de réduire les phénomènes inflammatoires et d´accélérer la résorption de l´ascite [4]. Chez la femme jeune le risque d´infertilité tubo-ovarienne est estimé à 39% [8].

Les limites de notre étude se résument surtout à la difficulté diagnostic vu la grande similitude clinique et para clinique avec le cancer de l´ovaire, la forme pseudo tumorale pose un véritable problème diagnostic différentiel avec la pathologie tumorale maligne souvent évoquée en premier devant la découverte d´une masse abdominale ou pelvienne qui évolue dans un contexte d´altération de l´état général [12]. Mais le point fort c´est que la quasi-totalité de nos patientes, et sous traitement anti bacillaire bien conduit après la chirurgie, ont eu une évolution favorable clinique et biologique comme ça était décrit dans la littérature [4].

## Conclusion

La tuberculose (TB) péritonéale est rare, son diagnostic est difficile en raison des nombreuses similitudes cliniques, biologiques et radiologiques avec la carcinose péritonéale. Elle devrait être évoquée devant toute masse abdominale ou pelvienne à caractère hyper vasculaire en particulier dans les zones d´endémie afin d´épargner à la patiente toute chirurgie d´exérèse inutile. Le recours aux explorations invasives est souvent inéluctable. En effet, en particulier dans les formes pseudo tumorales, seules la preuve histologique permet de réconforter le diagnostic.

### Etat des connaissances sur le sujet

- On sait déjà que La tuberculose péritonéale est très peu décrite dans la littérature avec un mode de révélation exceptionnel [1,2], sa symptomatologie clinique, radiologique et biologique simulant soit une tumeur ovarienne maligne soit un hydrosalpinx bilatéral associé à une ascite [13];
- L´imagerie est d´un grand apport pour le diagnostic positif grâce, essentiellement, aux prélèvements radioguidés [5];
- Une fois la preuve histologique obtenue, le traitement antibacillaire est souvent efficace avec une évolution clinique et radiologique spectaculaire [4].

### Contribution de notre étude à la connaissance

- Notre étude par rapport à la littérature est étalée sur une période de 10 ans avec une collecte patiente qui va permettre de comparer les différents tableaux cliniques, radiologiques et biologiques;
- En effet, s´il y a une chose que l´on devrait retenir de cette étude, ce doit être le grand polymorphisme clinique et radiologique de la tuberculose pelvienne pseudo-tumorale; il n´existe, jusqu´à maintenant, aucun marqueur biologique spécifique de cette pathologie et les prélèvements bactériologiques sont souvent négatifs et sources de retard diagnostique en raison de la lenteur des cultures;
- D´où l´intérêt indéniable de la laparoscopie, exploration qui permet non seulement d´accéder à la tumeur pour identifier sa nature et chercher des aspects macroscopiques associés évocateurs de tuberculose, mais habilite à réaliser des biopsies dans un but d´étude histo-bactériologique.

## References

[1] Hablani N, Souei Mhiri M, Tlili Graies K, Jemni Gharbi H, Abdallah S, Bel Hadj Hamida R (2005). La tuberculose abdominale pseudotumorale. J Radiol.

[2] Kefi A, Daoud F, Aydi Z, Baili L, Ben Dhaou B, Boussema F (2015). Granulomatose ovarienne tuberculeuse simulant un cancer de l´ovaire. La Revue de Médecine Interne.

[3] Lantheaume S, Soler S, Issartel B, Isch JF, Lacassin F, Rougier Y (2003). Tuberculose péritonéale disséminée simulant un cancer de l´ovaire: à propos d´un cas. Gynécologie Obstétrique et Fertilité.

[4] Abdallah M, Larbi T, Hamzaoui S, Mezlini E, Harmel A, Ennafaa M (2011). Tuberculose abdominale: étude retrospective de 90 cas. La revue de medecine interne.

[5] Eghdami L, Kwon J (2015). Tuberculose péritonéale se présentant sous forme d´une ascite persistante. J Obstet Gynaecol Can.

[6] Pommier J-D, Joly V (2016). Complications dans les suites d´une BCG thérapie intravésicale: épidémiologie, description clinique et prise en charge.

[7] Amouri A, Boudabbous M, Mnif L, Tahri N (2009). Profil actuel de la tuberculose péritonéale: étude d´une série tunisienne de 42 cas et revue de la littérature. La revue de médecine interne.

[8] Saadi H, Mamouni M, Errarhay S, Bouchikhi C, Banani A, Ammor H (2012). Tuberculose pelvi-péritonéale pseudotumorale: à propos de quatre cas. Pan African Medical Journal.

[9] Capron J, Lafont C, Grateau G, Steichen O (2010). Diagnostic non invasif d´une tuberculose péritonéale. La revue de médecine interne.

[10] Gobert D, Lidove O, Sicre de Fontbrune F, Peltier J, Chedid K, Burnat P (2007). Le dosage d´adénosine désaminase est utile pour le diagnostic de tuberculose péritonéale chez le patient dialysé. La revue de médecine interne.

[11] Grunenberger F, Schmitt V, Patry I, Buy X, Chenard MP, Schlienger JL (2009). Recherche d´une tuberculose par Quantiferon et lymphome non Hodgkinien. La revue de médecine interne.

[12] En-Nouali H, Seddik H, Elghari M, Salaheddine T (2008). Masse abdominale dans un contexte fébrile. Feuillets de Radiologie.

[13] Lahmiri M, Errabih I, Tamzaourte M, Krami HE, Benzzoubeir N, Ouazzani L (2012). Tuberculose péritonéale révélée par un tableau d´urgence chirurgicale. ALN éditions «Hegel».

